# Eye Tracking in Patients with Parkinson’s Disease Treated with Nabilone–Results of a Phase II, Placebo-Controlled, Double-Blind, Parallel-Group Pilot Study

**DOI:** 10.3390/brainsci12050661

**Published:** 2022-05-19

**Authors:** Philipp Ellmerer, Marina Peball, Federico Carbone, Marcel Ritter, Beatrice Heim, Kathrin Marini, Dora Valent, Florian Krismer, Werner Poewe, Atbin Djamshidian, Klaus Seppi

**Affiliations:** 1 Department of Neurology, Medical University Innsbruck, Anichstraße 25, 6020 Innsbruck, Austria; philipp.ellmerer@student.i-med.ac.at (P.E.); marina.peball@i-med.ac.at (M.P.); federico.carbone@student.i-med.ac.at (F.C.); beatrice.heim@i-med.ac.at (B.H.); kathrin.marini@student.i-med.ac.at (K.M.); dora.valent@student.i-med.ac.at (D.V.); florian.krismer@i-med.ac.at (F.K.); werner.poewe@i-med.ac.at (W.P.); atbin.djamshidian-tehrani@i-med.ac.at (A.D.); 2 Interactive Graphics and Simulation Group, University of Innsbruck, Innrain 52, 6020 Innsbruck, Austria; marcel.ritter@uibk.ac.at

**Keywords:** Parkinson’s disease, nabilone, cannabis, eye-tracking, non-motor symptoms

## Abstract

The topic of the therapeutic use of cannabinoids in Parkinson’s disease (PD) is broadly discussed and frequently comes up in the outpatient clinic. So far, there are only a few randomized clinical trials assessing the effects of cannabinoids in PD. We are able to demonstrate a reduction in non-motor symptom (NMS) burden after the administration of nabilone. As impairment of attention and working memory have been described earlier as possible side effects, we assess cognitive performance using saccadic paradigms measured by an eye tracker. We do not observe a significant difference in any of the saccadic paradigms between PD patients on placebo versus those treated with nabilone. We, therefore, conclude that top-down inhibitory control is not affected by the tetrahydrocannabinol analogue. Nabilone did not significantly worsen cognitive performance and appears to be safe to use in selected PD patients who suffer from disabling NMS.

## 1. Introduction

The potential therapeutic effect of cannabinoids in Parkinson’s disease (PD) is a prominent topic and commonly raised by patients in the consulting room, but the evidence is still limited in supporting their use in PD due to a paucity of sufficient clinical data about their efficacy and safety in PD patients [1,2]. Recently, the synthetic tetrahydrocannabinol analogue nabilone has been shown to improve overall non-motor symptom (NMS) burden, especially reflected by amelioration of anxiety and sleeping problems [3].

However, raised concerns about potential cognitive side effects such as impairment of attention as well as working and episodic memory have been described in the past in patients treated with cannabinoids [4]. Moreover, conceptual disorganization, depersonalization and derealization, distorted sensory perceptions [5], and rarely abuse, and tolerance after tetrahydrocannabinol and nabilone intake have been observed [4].

Eye-tracking provides an innovative, broadly available, and relatively cheap option in order to assess cognitive functions such as attention, emotion recognition, or inhibitory control. It has been broadly used in various neurologic and psychiatric conditions such as PD, Alzheimer’s disease, cervical dystonia, Restless Legs Syndrome, as well as in schizophrenia or autism, and has been proposed as a method to track cognition in progressive neurodegenerative conditions [6,7,8,9]. It has been shown in previous studies that oculomotor performance is generally stable over time. Saccadic tasks show very good internal reliability [10]. Furthermore, the test-retest reliability for saccadic tasks shows good to excellent reliability for reaction times and error rates [11].

Therefore, we added eye-tracking as an exploratory endpoint of the phase two placebo-controlled double-blind parallel-group pilot study, using an enriched enrolment randomized withdrawal (EERW) design. We wanted to evaluate cognitive functions such as working memory, attention, and inhibitory control, measured in response times and error rates. Furthermore, we wanted to assess whether nabilone would cause impairment in cognitive performance in patients with PD.

## 2. Materials and Methods

We refer to the study design paper [1] as well as the primary outcome of the study [3] for a detailed description.

**Figure 1:**
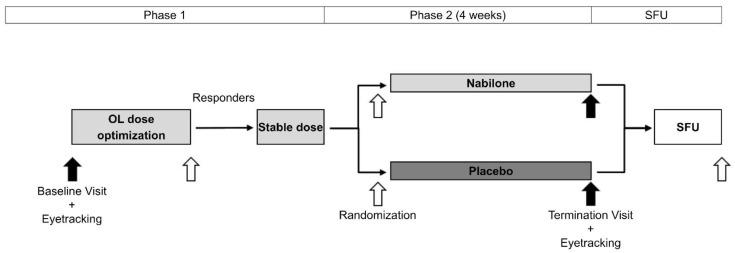
Schedule of trial activities. Visits are marked with arrows. Timepoints with eye-tracking analysis are marked with black arrows. OL…Open-label; SFU…Safety follow-up. Modified Figure from “Non-Motor Symptoms in Parkinson’s Disease are Reduced by Nabilone” by Peball et al., published in Annals of Neurology 2020, 88, pp. 712–722 [3].

Briefly, the NMS-Nab Study was a mono-centric phase II, randomized, placebo-controlled, double-blind, parallel-group, enriched enrollment withdrawal study in patients with a variety of NMS in PD. A sample of 47 patients with PD with stable motor disease and disturbing NMS defined by a score of ≥4 points on the Movement Disorder Society-Unified PD Rating Scale-I (MDS-UPDRS-I) was included in this study (Figure 1). At the baseline visit open-label nabilone was started with a dosage of 0.25 milligrams (mg) in the evening. During dose titration period, nabilone was titrated in 0.25-mg increments once or twice daily until a maximum dosage of 1 mg twice daily. After this titration phase (phase 1), subjects were either considered responders according to change in the Clinical Global Impression of Improvement Scale (CGI-I) or discontinued from the study (n = 9). The remaining 38 participants were randomized to either optimal nabilone dose or matching placebo (phase 2). After the 4-week, double-blind, withdrawal phase, a termination visit was performed.

Eye-tracking was added as an exploratory outcome measure after study initiation of the original protocol as an amendment in January 2018. Therefore, only a total of 26 out of the 38 patients could be included in this eye-tracking analysis. All subjects performed a Montreal Cognitive Assessment (MoCA), the MDS-UPDRS parts I-IV, in addition a Mini-Mental State Examination (MMSE,) and an eye-tracking battery at the baseline visit as well as at the termination visit (after a 4-week double-blind phase). Eye-tracking was only performed twice in order to minimize learning effects.

Eye movement data were recorded using the Tobii TX-300 eye tracker and the Tobii Pro Lab Software (v.1.83). A sampling rate of 300 Hz was used. Before every task, a 9-point-calibration was done in order to assure a gaze position accuracy of less than 0.4°. Subjects were seated at a 64 cm distance from the presentation screen. The eye-tracking assessment consisted of a prosaccade task, an antisaccade task, a mixed (Go/-NoGo) task, and a mixed pro-, antisaccade task. The centralized fixation cross and the peripheral cues were 0.4° in size. The cues were placed approximately 10° horizontally to each side. The background of the display was set to black and cues were presented in white or in some cases colored depending on the task, in order to achieve maximum contrast. All tests were pseudorandomized in order to assure that all participants experienced the same order of trials. Prior to every task, subjects were orally and visually instructed and performed four test runs.

The prosaccade task consisted of 80 trials in total (40 on each side). A centralized fixation cross (white) appeared on screen. We used an overlap design. Therefore, the peripheral cue appeared after a delay and patients were instructed to look at the peripheral cue only after the central fixation cross disappeared. In order to minimize habituation, we used different delays (500 ms vs. 700 ms).

Secondly, all participants performed an antisaccade task with a step design. The assessment consisted of 20 trials. Cues used were the same as in the prosaccade task (central fixation cross [1500 ms], peripheral round cue [1000 ms], both approximately 0.4° size).

Then, all patients were asked to perform a Go/NoGo task (stop saccades), consisting of 60 trials in total. In this test the direction of the appearing cue was indicated in advance by a flashing green arrow (100 ms). However, in 30% of trials, a red stop signal (200 ms) appeared after the green arrow. In this case, participants were asked to suppress the prosaccade towards the cue. No gap was used.

Lastly, all participants performed a mixed pro- and antisaccade task. The centralized fixation cross (1000 ms) would change color into either blue or green (500 ms), indicating the saccade that had to be initiated by the subject. The test consisted of 40 trials (28 prosaccades, 12 antisaccades). This trial was then repeated with the colors being switched.

Artifact correction and calculation of saccade parameters were carried out by the use of custom-developed interactive analysis software. Reaction times were calculated by the use of linear regression as well as eye-movement interval classification. The automated detection was manually corrected and adjusted if necessary and exported to Microsoft excel files for further analysis. Saccade initiation was defined as an eye movement greater than 2° and a velocity greater than 30°/s. Trials where evaluation was not possible due to a lack of data quality (blinks, eye recognition errors, etc.) were excluded from analysis. Reaction times in milliseconds (ms) were defined by the latency between stimulus onset and saccadic initiation. Furthermore, error rates (%) were calculated. Errors were defined as premature saccades (latency below 70 ms), direction errors, and failure to initiate a saccade. In the Go/NoGo paradigm, saccades that should have been suppressed were considered errors as well.

The study (NMS-Nab Study, EudraCT 2017-000198-86) was approved by the Research Ethics Committee of Innsbruck Medical University, Austria, before study initiation (reference number 1008/2017). All participants provided written informed consent according to the Declaration of Helsinki.

Statistical analysis was carried out using SPSS Statistics 26. Parametric or nonparametric tests were used for statistical analysis depending on the scale type and data distribution. Quantitative values are given in mean ± SD or median and interquartile range. For evaluation of the eye-tracking parameters, a repeated-measures ANOVA was calculated. For variables not meeting the criterion of normal distribution, logarithmic transformation or the min-max method were applied. The level of significance was set at *p* < 0.05.

## 3. Results

A total of 24 subjects performed the eye-tracking assessment battery in both visits with sufficient data quality. Two subjects were excluded due to insufficient calibration results. There were no significant differences in demographic characteristics at baseline (Table 1).

Results of the eye-tracking assessment are presented at Table 2 and graphically in the supplementary material (Figure S1).

We found a significant difference in the error rate of the antisaccade task between the baseline and the termination visit. Both patient groups (nabilone vs. placebo) made fewer errors at the termination visit compared to the screening visit (*p* = 0.046). There was no significant difference in the reaction time between the baseline and the termination visit (*p* = 0.070).

There were no significant group differences between the placebo and the nabilone groups in the reaction times of the other saccadic paradigms (prosaccade task, antisaccade task, countermanding task, and pro-, antisaccade task) (*p* > 0.1). Furthermore, there was no difference in error rates between the placebo and the nabilone groups (*p* > 0.1). There was also no difference between the baseline and termination visits in the MoCA score (*p* > 0.07).

## 4. Discussion

In this preliminary study, we report no difference in any of the saccadic paradigms between PD patients on placebo versus those who took nabilone. More specifically, there was no change in several tasks that require top-down inhibitory control that relies on intact frontal area circuitry (particularly the dorsolateral prefrontal cortex, frontal eye fields, and the superior colliculus). Furthermore, there was no change in visual fixation and saccadic control that required a wide network including the thalamus, the cerebellum, the basal ganglia, the parietal eye field, and the brainstem [12], and there was no difference in the MoCA score between the two groups. However, we found a significant difference in error rates between the baseline and the termination visit, suggesting that there was a learning effect in both patient groups. This implies that nabilone also does not impair the ability to learn and consolidate new information.

Interestingly, we found similar error rates in the prosaccade and antisaccade tasks. Importantly, however, we did not use a simple step design for the prosaccade task but a step-over design. This specific design requires a higher top-down inhibitory control and may therefore explain our findings.

This is of special interest as the cannabinoid system plays an important role in cognitive function. CB1 receptors are expressed in various brain regions, including the prefrontal cortex, basal ganglia, and hippocampus [13], and play, in combination with the dopaminergic system, an important role in memory consolidation [14,15]. Moreover, the endocannabinoid system regulates working memory processes and modulates the circuits between the hippocampus and the prefrontal cortex [16]. Acute cannabis intoxication is well known to cause cognitive dysfunction [17], but the effects of the endocannabinoid system on cognitive performance are likely dose-dependent. In line with this, a previous study has shown the beneficial effects of cannabis on memory functions in PD [18]. Therefore, it is possible that the median dosage used in our study was below the threshold and did not affect reaction times, learning, and task performance overall. Future studies with larger sample sizes are needed in order to assess potential dose-dependent effects.

Top-down prefrontal cortex inhibitory control, which is necessary for the tasks used here [9], is likely also related to the dopaminergic system [19]. It is well known that patients with dyskinesias have an impairment in tasks that require inhibitory control and score higher on self-reported impulsivity questionnaires [20]. Interestingly, nabilone has the potential to reduce dyskinesias in PD, probably due to a reduction of GABA in the globus pallidus without worsening the antiparkinsonian effects of dopaminergic therapy [21].

Nabilone also plays a role in patients with drug addiction [22,23] and has been shown to reduce agitation in patients with Alzheimer’s disease [24]. Whether nabilone can indeed reduce impulsivity or drug craving in PD remains unclear and is beyond the scope of this study.

However, there are a few limitations to our study. Firstly, as eye-tracking was added as an exploratory endpoint by an amendment to the main study, not all PD patients included in the main study underwent the eye-tracking examination. Nevertheless, we could include 24 patients in this exploratory analysis. Secondly, eye-tracking was not performed at all visits. As learning effects in saccadic paradigms have been described earlier [25], we limited eye-tracking examinations to the baseline visit when open-label nabilone was started and at the termination visit, which was the last visit of the randomized, placebo-controlled wash-out phase. Performing the second eye-tracking examination at the end of the wash-out period assured the longest time possible between the eye-tracking examinations. Furthermore, eye-tracking is not a gold standard for examination in order to assess cognition. However, it has been extensively used in various neurologic and psychiatric conditions, including cognitive dysfunction [6]. Moreover, we additionally used the MoCA at the baseline and termination visit, which also did not show a significant group difference. Although we applied the eye tracking examinations with the longest possible interval in between, we detected a group difference in the antisaccade error rate. Our findings may be caused by learning effects within the antisaccade paradigm. This is in accordance with a previous study assessing the reliability of saccadic paradigms, where effects of practice were especially prominent in the antisaccade task. Participants performed better as measured by a reduced error rate and improved spatial accuracy at retest [10]. Lastly, five patients were classified as non-responders in the open-label titration phase and discontinued the study. Therefore, these patients were not included in the double-blind washout phase, which may have had an impact on our findings. This is one of the disadvantages of using this trial design [3].

Nevertheless, we did not observe a significant difference in any of the saccadic paradigms between PD patients on placebo versus those treated with nabilone. Thus, in our preliminary study, nabilone did not worsen ocular motility or saccadic inhibitory control. However, due to the small sample size and the short observation period, the results should be interpreted with caution. Studies with a larger sample size and a longer observation period are needed to replicate our preliminary results.

## Figures and Tables

**Table 1:** Demographics. MMSE…Mini Mental State Examination; MoCA…Montreal Cognitive Assessment; HADS…Hospital Anxiety and Depression Scale; MDS-UPDRS…Movement Disorder Society Unified Parkinson’s disease Rating Scale; a…Student’s *t*-test; b…Welch’s *t*-test; c…Wilcoxon rank-sum test; d…Chi-squared test.

	Nabilone	Placebo	*p* Value
n (f)	14 (6)	10 (1)	0.081 ^d^
Age (y)	65.9 ± 7.5	62.9 ± 9.3	0.376 ^a^
Disease duration (y)	7.0 ± 5.7	5.4 ± 2.0	0.347 ^a^
Education (y)	12.3 ± 2.6	13.3 ± 3.0	0.391 ^b^
MMSE	30.0 (29.0–30.0)	29.5 (29.0–30.0)	0.503 ^c^
MoCA	28.0 (27.0–29.75)	28.0 (27.0–29.0)	0.928 ^c^
HADS-A	6.6 ± 3.6	4.3 ± 2.8	0.099 ^a^
HADS-D	5.6 ± 3.6	4.2 ± 2.7	0.296 ^a^
MDS-UPDRS I	13.9 ± 4.4	11.2 ± 4.5	0.152 ^a^
MDS-UPDRS II	10.9 ± 6.3	10.8 ± 4.1	0.956 ^a^
MDS-UPDRS III	25.9 ± 12.7	30.3 ± 11.4	0.395 ^a^
MDS-UPDRS IV	1.0 (0.0–3.0)	1.5 (0.0–3.8)	0.900 ^c^

**Table 2:** Eye-tracking tasks: BL…Baseline visit; TV…Termination visit; PS…Prosaccades; AS…Antisaccades; SS…Stop saccades; PAS…Pro-, Antisaccade task; RPAS…Reversed pro-, antisaccade task; RT…Reaction time (ms); ER…Error rate (%); M…Mean; SD…Standard deviation; a…Data has been normalized by logarithmic transformation; b…Data has been normalized by the minimum-maximum method; *p*-values numbers marked in bold indicate numbers that are significant.

	Nabilone		Placebo		Main Effect Time	Main Effect Group	Main Effect Time × Group
	M	SD	M	SD	*p*	*p*	*p*
PS RT (ms) ^a^							
BL	277	110	300	67	0.766	0.799	0.158
TV	297	81	287	124			
PS ER (%) ^a^							
BL	59.5	31.8	59.8	33.6	0.484	0.893	0.292
TV	49.7	28.9	58.2	35.5			
AS RT (ms) ^a^							
BL	295	110	302	83	0.070	0.763	0.705
TV	327	82	330	90			
AS ER (%)							
BL	58.7	27.8	46.7	37.4	**0.046**	0.215	0.539
TV	51.8	29.6	34.1	26.1			
SS RT (ms) ^a^							
BL	227	46	246	72	0.248	0.514	0.941
TV	216	77	231	51			
SS ER (%)							
BL	28.25	19.95	31.78	26.62	0.137	0.723	0.109
TV	28.73	20.85	19.28	19.15			
PAS RT (ms) ^a^							
BL	264	65	237	48	0.133	0.525	0.313
TV	269	53	271	88			
PAS ER (%)							
BL	25.5	15.5	25.0	20.0	0.757	0.831	0.455
TV	29.4	22.9	25.0	20.7			
RPAS RT (ms) ^a^							
BL	231	51	237	43	0.188	0.912	0.748
TV	243	50	237	51			
RPAS ER (%) ^b^							
BL	27.7	14.2	27.5	21.7	0.735	0.85	0.16
TV	29.4	14.7	21.6	25.0			

## Supplementary Materials

The following supporting information can be downloaded at: https://www.mdpi.com/article/10.3390/brainsci12050661/s1, Figure S1: Results of eye tracking tasks.
