# Supplementary material for: Eye Tracking in Patients with Parkinson’s Disease Treated with Nabilone–Results of a Phase II, Placebo-Controlled, Double-Blind, Parallel-Group Pilot Study
Source: Brain Sci. 2022 May 19;12(5):661. doi: 10.3390/brainsci12050661 (PMC9139535; doi:10.3390/brainsci12050661)
Supplement: Supplementary file 1 [file brainsci-12-00661-s001.zip › brainsci-1524066-supplementary.pdf]

Supplementary Materials:

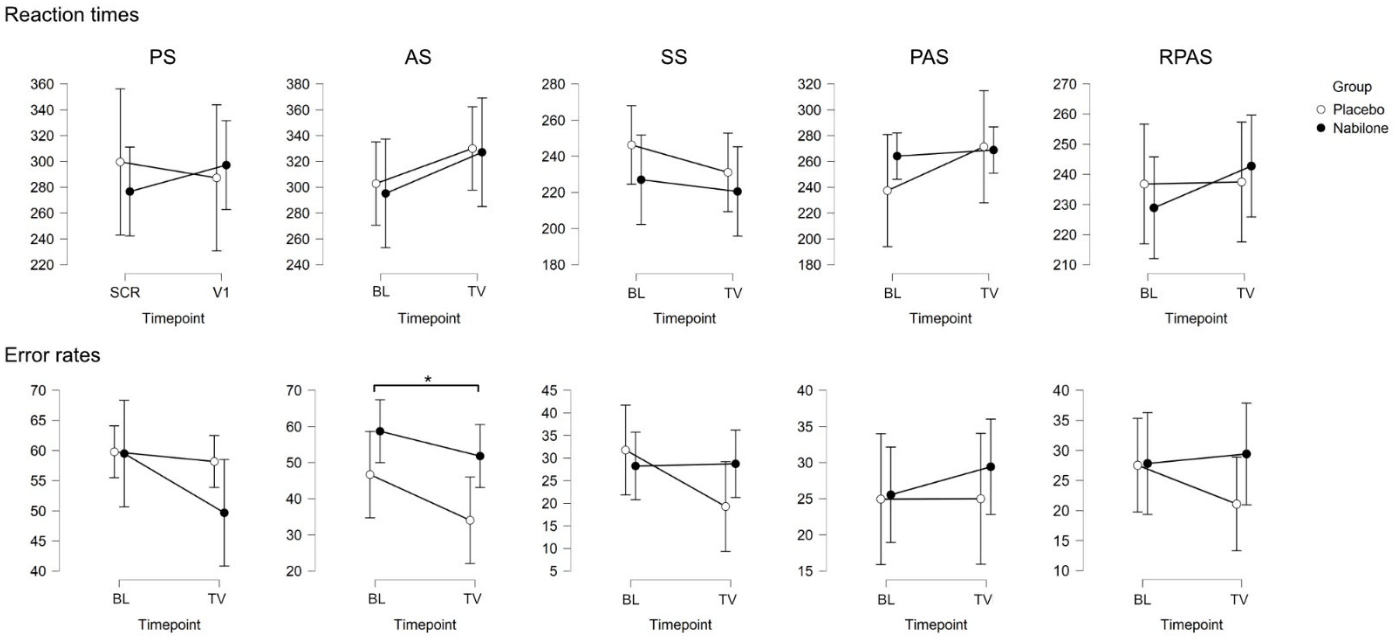

Figure S1. Results of eye tracking tasks. PS…Prosaccades; AS…Antisaccades; SS…Stopsaccades; PAS…Pro/Antisaccades; RPAS; Reversed Pro/Antisaccades; BL…Baseline visit; TV…Termination visit; Reaction times given in ms; Error rates given in percentage; Plots show mean and the 95% confidence interval; Asterisks indicate statistically significant differences.
